# Stress matters! Psychophysiological and emotional loadings of pregnant women undergoing fetal magnetic resonance imaging

**DOI:** 10.1186/s12884-015-0448-9

**Published:** 2015-02-13

**Authors:** Birgit Derntl, Jacqueline Krajnik, Kathrin Kollndorfer, Manfred Bijak, Ursula Nemec, Katharina Leithner, Daniela Prayer, Veronika Schöpf

**Affiliations:** Department of Psychiatry, Psychotherapy, and Psychosomatics, RWTH Aachen University, Pauwelstrasse 30, 52074 Aachen, Germany; Jülich Aachen Research Alliance JARA BRAIN, Translational Brain Medicine, Jülich, Germany; Institute for Neuroscience and Medicine (INM-1), Research Center Jülich, 520425 Jülich, Germany; Department of Biomedical Imaging and Image-guided Therapy, Medical University of Vienna, Währinger Gürtel 18-20, 1090 Vienna, Austria; Center for Medical Physics and Biomedical Engineering, Medical University of Vienna, Währinger Gürtel 18-20, 1090 Vienna, Australia; Department of Psychoanalysis and Psychotherapy, Medical University of Vienna, Währinger Gürtel 18-20, 1090 Vienna, Australia

**Keywords:** Fetal MRI, Stress, Pregnancy, Emotional well-being

## Abstract

**Background:**

While the application of fetal MRI in high-risk pregnant women is steadily rising, little is known about the psychological consequences of this procedure. The aim of the present study was to investigate emotional and psychophysiological reactions of females undergoing fetal MRI.

**Methods:**

Sixty women (17–44 ys), assigned for fetal MRI, were included. Affective state was assessed by standardized measures of anxiety, emotional states and depressive symptoms. Stress coping strategies were assessed using a self-report questionnaire. Stress responses were determined using skin conductance levels (SCL) during fetal MRI as well as measurement of salivary cortisol levels immediately before and after fetal MRI.

**Results:**

Analysis of fast and slow physiological stress measures revealed significant differences between women with and without a supporting person accompanying them to the examination. For SCLs, lower levels of stress during MRI emerged in accompanied women. Women with well-marked stress-coping-strategies experienced lower levels of stress during the examination. Although fast and slow stress measures before and after MRI did not show significant correlations, a significant difference of SCLs pre and post examination was clearly detectable, as well as a trend of decreased cortisol levels for both time points.

**Conclusions:**

The results imply that the elevation of SCLs is an accurate instrument to assess fast stress alterations in patients during fetal MRI. Stress coping strategies and whether women are accompanied or not play an important role in the experience of anxiety and depressive symptoms. These factors should be considered especially in patients with high-risk-pregnancies to improve patient care.

## Background

Fetal magnetic resonance imaging (MRI) is increasingly used in prenatal diagnosis and can provide additional information superior to routine ultrasonography [1,2]. Pregnant women undergoing MRI exams often receive general information on the procedure, however have limited understanding of what to expect during the examination [3]. Hence, this implementation of highly developed prenatal diagnostic methods may cause insecurity, stress, and anxiety in pregnant women [4,5]. Previous studies have shown that anxiety and insecurity are influenced by the state of pre-information and the patient’s experiences of the MRI examination. Particularly sufficient pre-information regarding the examination procedure and the expecting experiences given by physicians are of great importance and may help to ease the women’s anxiety for MRI exams [4,6].

Stress reactions are normally evoked in uncontrollable and unpredictable situations and physical reactions accompanying these reactions, such as increased heart rate or a higher level of vigilance, are part of an automated response providing assistance in managing extreme and demanding situations (for review see [7]). Threatening or more demanding stressful situations can eventually lead to feelings of anxiety as stress and anxiety are highly interactive (e.g., [8,9]). The physiological bases of any stress response are the hypothalamic-pituitary-adrenal (HPA) axis and the sympathetic adrenomedullary system (SAM), which is part of the autonomic nervous system (ANS).

A permanently increased stress response is considered to be a potential risk factor for coronary heart diseases specifically in males [10,11], while in females chronic stress rather leads to depression and anxiety [12,13].

One group with potentially high stress reactions are women with high-risk pregnancies [4]. In this group, stress responses not only affect emotional well-being of the patients but also interact with the progression of stress-related physiological responses, probably inducing long-term effects on the offspring’s postnatal development. Maternal stress hormones carry information about the external world, helping the fetus to prepare for the environmental situation after birth [14]. Thus, the maternal stress hormone concentration provides significant information to the offspring. However, chronic stress during pregnancy should be avoided, as it is considered to negatively influence the fetal development. Maternal prenatal stress has been associated with low birth weight [15], preterm birth [16,17], cognitive development [18,19] as well as mental or behavioral disorders such as schizophrenia [20] or attention deficit hyperactivity disorder [21].

The aim of the present study was to assess the psychophysiological and emotional reactions of women with high-risk pregnancies undergoing an MRI examination. We particularly focused on the stress level before, during and after the MRI examination, which was assessed using two different methods: salivary cortisol and skin conductance levels (SCL). Salivary cortisol levels represent stress reactions activating the HPA axis, whereas skin conductance is a measure of the activation of the ANS. Additionally, behavioral data regarding depressive symptoms, state and trait anxiety, mood states, and stress coping strategies were collected. Previous studies have shown that MRI examinations in pregnant women are perceived as a very stressful situation by the mothers (cf. [4]). Thus, a particular aim of this study was the investigation of psychophysiological parameters that might give insight in how these female patients react to and cope with this situation, thereby helping to reduce the stress experience and anxiety levels to significantly improve patient comfort during the MRI examination.

## Methods

### Subjects

Sixty-three pregnant women who were referred from different gynecological practices situated in and around Vienna as well as from the Department of Gynecology of the Medical University of Vienna, Austria, underwent fetal MRI at the Department of Biomedical Imaging and Image-guided Therapy, Medical University of Vienna. Three subjects had to be excluded due to an early drop out. Therefore, data from 60 females between the age of 17 and 44 years (mean 29.97; SD 6.3) and gestational week 16 to 37 (mean 26.52; SD 4.7) at the time of fetal MRI were included. None of the females reported having suffered from neurological, psychiatric or psychosomatic diagnoses and none was taking medication. Additionally, we assessed how referred mothers were informed about the fetal MRI examination (for more information see Table 1). Five females out of the 63 mothers have already had a fetal MRI examination due to a previous pregnancy.

**Table 1:** **Descriptive information on how mothers were informed about the fetal MRI measurement prior to their visit**

			**MRI leaflet**		
			**No**	**Yes**	**Total**
Information from referring physician	No	Count	8	4	12
		% of total number	13.3%	6.7%	20.0%
	Yes	Count	23	25	48
		% of total number	38.3%	41.7%	80.0%
Total		Count	31	29	60
		% of total number	51.7%	48.3%	100.0%

Regardless of the information status, mothers received information on the procedure by a medical professional immediately before the scanning session.

### Ethics statement

All participants were informed about the aim of the study and gave their written informed consent prior to inclusion. The study was performed in accordance with the Declaration of Helsinki (1964), including current revisions and the EC-GCP guidelines and was approved by the Ethics Committee of the Medical University of Vienna (026/2006).

### Data acquisition

All measurements were performed on a 1.5 T system (Philips Gyroscan, Philips, Best, The Netherlands) using a five-channel phased-array surface coil. Imaging included the following sequences: steady-state free-precession (SSFP) surveys, reference scans, T2-weighted TSE sequences, T1-weighted sequences, SSFP and dynamic SSFP sequences, echo planar sequences (EPI), diffusion weighted imaging (DWI), diffusion tensor imaging (DTI), fluid attenuated inversion recovery (FLAIR) sequences, and single voxel spectroscopy. Mean duration of examination did not exceed 45 minutes [22].

Subjects were classified due to the severity of previous ultrasound using three different categories: no fetal malformation (group 1), fetal pathology compatible with survival (group 2), and fetal pathology probably not compatible with survival (group 3) [6] (group 1: 10 subjects, no fetal malformation; group 2: 35 patients in total, mildly enlarged ventricles (no identifiable cause, n = 9), enlarged cisterna magna (no identifiable cause, n = 2), mild IUGR (n = 5), skeletal pathologies (cleft lift and palate (n = 4), clubfood (n = 1), micorcephaly (n = 1)), unilateral kidney pathologies (hydronephrosis (n = 4), multicystic kidney (n = 1), diaphragmal hernia (contralateral lung volume normal, n = 2), CCAM (n = 2), gastroschisis (n = 2), partial CCA (n = 1), polyhydramnios (no identifiable cause, n = 1); group 3: 15 patients in total, large menigomyelocele (n = 1), complex congenital heart defects (n = 6), stillbirths (n = 4), large fetal tumors (n = 2), massive hydrocephalus (n = 1), large spina bifida (n = 1)).

**Figure 1:**
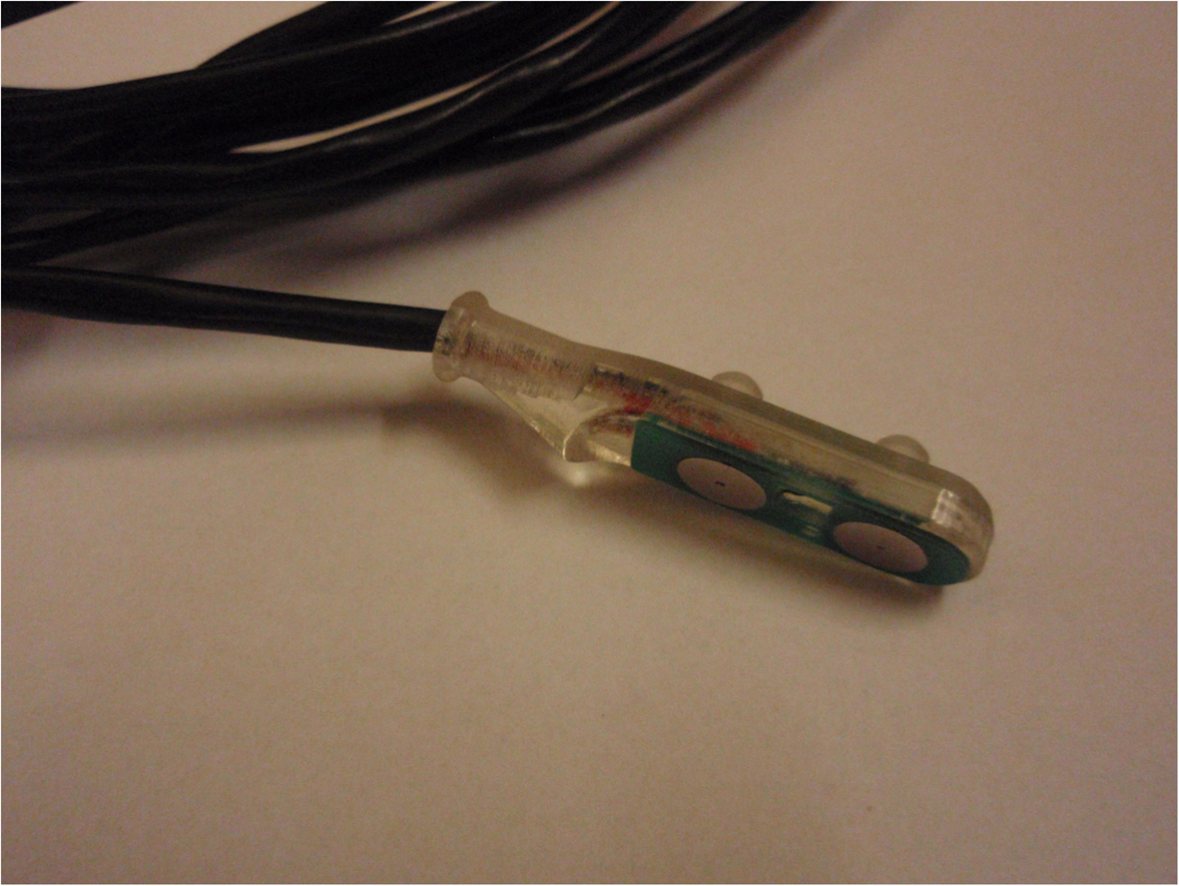
**Non-polarizing MR compatible electrode with an eight meter twisted pair cable used for recording skin conductance levels of subjects while lying in the scanner.**

Saliva samples were collected before and after the MRI examination for cortisol measurements. Skin conductance levels were monitored and recorded during the MRI session using a non-polarizing MR compatible electrode (see Figure 1). The electrode was placed on the second finger of the non-dominant hand of the subject using adhesive tape. An eight meter twisted pair cable was used to connect the electrode with the recording unit outside the scanner room. The SCL measurement devices were manufactured in cooperation with the Center for Medical Physics and Biomedical Engineering of the Medical University Vienna.

**Figure 2:**
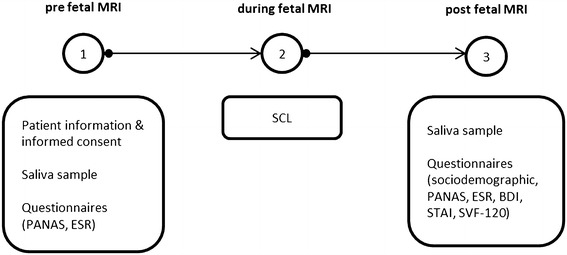
**Graphic representation of the experimental design for all subjects.** Measurements for each subject took place on the same day (between 7 and 9 am) and included three different subprocedures. Immediately before fetal MRI (1) patients were informed about the whole experiment, gave their written informed consent and completed two questionnaires. Subsequently saliva samples were taken. During fetal MRI (2) skin conductance levels were monitored and recorded. Immediately after fetal MRI (3) saliva samples were taken, and several questionnaires were completed.

Mood states were tested using PANAS scales [23] and self-ratings of fear were assessed before and after the MRI examination. Moreover, after completing the MRI examination and assessment of socio-demographic and pregnancy-related data, all subjects underwent neuropsychological testing, including questionnaires tapping anxiety (State-Trait-Anxietyinventory STAI; Laux et al., [24]), depressive symptoms (Beck Depression Inventory-II, BDI-II [25]), and stress coping strategies (Stressverarbeitungsfragebogen, SVF 120; [26]). A schematic visualization of the data acquisition process can be found in Figure 2.

### Data analysis

All participants arrived between 7 and 9 am. We obtained two saliva samples, the first immediately before and the second immediately after fetal MRI. Cortisol samples were analyzed at the SwissHealthMed laboratory (Aying, Munich, Germany). Upon arrival of the samples in the analysis laboratory the samples were frozen at −20°C at least overnight. To precipitate mucins, samples were thawed and centrifuged at 3000–2000 × g for 10 min. Competitive Luminescence Immunoassay kits (LUMI) were used to measure concentration of cortisol (pg/ml). The LUMI kit is based on the competition principle. These kits have minimal cross-reactivity to other steroid hormones. Measurements were highly reliable (intra-assay CV < 4% and inter-assay CV < 5%). The lower limit of sensitivity of the immunoassay kit was 0.003 μg/dL for cortisol.

Skin conductance time course measures were processed using Matlab 7.8.0 (R2000a). Analysis included time course characteristic calculation of mean difference of the first and last five minutes.

Statistical analyses were performed using the Statistical Package for the Social Sciences, Version 20.0 (SPSS, Chicago, Illinois). The alpha level for all tests was set at p = 0.05. For significant effects partial eta-squared (η_p_^2^) are listed as estimates of effect size.

## Results

Due to technical matters (broken finger electrode) SCL measures were acquired from 37 females. Moreover, due to insufficient amount of saliva, complete saliva samples for cortisol analyses were collected from 58 females. Saliva and SCL measures from two females had to be excluded due to nausea during the MRI examination. No significant correlation was obtained for SCL and cortisol levels (r = −0.018; p = 0.922). Subjects showed significant higher cortisol levels immediately before fetal MRI examination (t = 4.215, p < .001), as well as significant higher skin conductance levels during the first five minutes in the MR scanner (t = 2.733, p = 0.010 (see Table 2)).

**Table 2:** **Description of cortisol levels immediately before (t1) and after (t2) fetal MRI examination and for skin conductance levels of the first 5 minutes (t1) and last 5 minutes (t2)**

	***N***	***Mean***	***SD***
*Cortisol t1 (pg/ml)*	56	6415.36	2974.14
*Cortisol t2 (pg/ml)*	56	5182.32	2169.01
*SCL t1 (μs)*	35	29644.14	12786.42
*SCL t2 (μs)*	35	25369.11	12627.83

Mean values and standard deviation for cortisol levels, as well as for SCL measures, and number of subjects are included. Cortisol levels (t = 4.215, p < .001) and SCL values (t = 2.733, p = .010) differed significantly between t1 and t2.

Correlation analysis revealed a significant association of age with depressive scores of participants and cortisol levels measured after fetal MRI scan (see Table 3).

**Table 3:** **Representation of detected significant correlations**

	**Correlation coefficient (r)**	**Significance (p)**
**Depressive score (BDI)**	0.292	**0.024**
**Cortisol (t2)**	−0.274	**0.037**

Correlation analysis revealed significant results for SCL-differences, depressive score (BDI) and cortisol (t2) and confounder variables gestational week and age.

Based on these results the impact of accompanying person, diagnosis and stress management strategies (for further group description see Table 4) were analyzed on cortisol levels, SCL measures, and psychological parameters (mood, depression, anxiety). Results are reported separately in the following paragraphs.

**Table 4:** **Different categorizations of all 60 subjects were performed ahead of statistical analysis**

***Groups***	***N***	***Age (mean)***	***GW (mean)***
*Accompanying person*			
Yes	35	30.8	26.5
No	25	29.3	26.5
*Prenatal Diagnosis*			
No fetal malformation (1)	10	35.3**	25.2
Fetal pathology compatible with survival (2)	35	29.1	27.0
Fetal pathology probably not compatible with survival (3)	15	28.4	26.3
*Stress-regulation-strategy*			
Below average strategies	11	29.3	26.0
Average strategies	30	30.2	26.5
Above average strategies	19	30.0	26.9

An overview of these three groups and inherent subgroups is given in this table indicating number of subjects (N), mean age and mean gestational week (GW). For the variable accompanying person groups neither differed in age (p = 0.363) nor gestational week (p = 0.996) or diagnosis. Analysis for prenatal diagnosis revealed no difference in gestational week (F(2,57) = 0.573, p = 0.567); but patients in group 1 were significantly older compared to both other groups, (1 vs. 2: p = 0.008; 1 vs. 3: p = 0.001), while group 2 and 3 did not differ (p = 0.703). For stress-regulation strategy groups neither differed in age (F(2,57) = 0.129, p = 0.879) nor gestational week (F(2,57) = 0.083, p = 0.920). Significant differences are marked in the table with **.

### Accompanying person

We divided our participants into accompanied (n = 35) or non-accompanied (n = 25) females. Groups neither differed in age (p = 0.363) nor gestational week (p = 0.996) or diagnosis (p = 0.096; see also Table 4).

#### Cortisol

**Figure 3:**
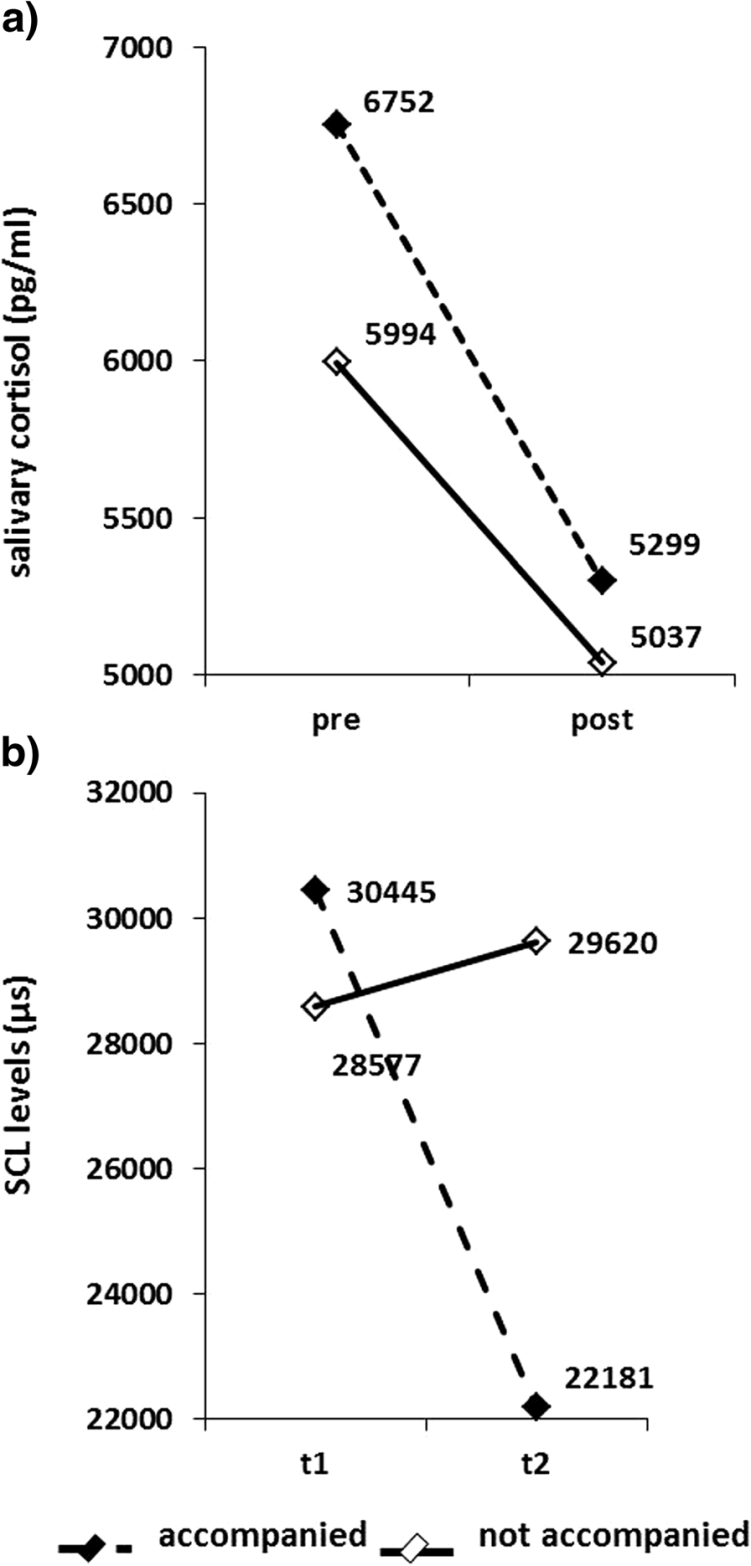
**Stress measures cortisol and skin conductance measures before and after fetal MRI depending on accompanying person. a)** All subjects showed higher cortisol levels before compared to post examination. **b)** Skin conductance levels (SCL, in μs) of the first 5 minutes (t1) and last 5 minutes (t2) of fetal MRI examination of women with (yes) and without (no) support by an accompanying person. Accompanied women showed a significant reduction of SCL-levels at the last 5 minutes of the examination.

Due to significant influence of the variable age (r = −0.274; p = 0.037), changes in cortisol levels were analyzed using an analysis of covariance for repeated measurements with the within-subject factor time (pre and post MRI) and the between-subject factor group (with or without accompanying person). Analysis revealed no significant main effect of time (F(1,53) = 0.522, p = 0.473), no significant group effect (F(1,53) = 0.629, p = 0.431), and no significant time-by-group interaction for cortisol levels (F(1,53) = 0.710, p = 0.403) emerged (see Figure 3a).

#### SCL

Repeated measurements analysis of variance (rmANOVA) with the within-group factor time (t1, t2) and between-subjects factor group (with or without accompanying person) revealed a significant time effect (F(1, 33) = 6.798, p = 0.014, η_p_^2^ = 0.171) with higher values at t1, no significant group effect (F(1, 33) = 0.467, p = 0.499) but a significant time-by-group interaction (F(1, 33) = 11.294, p = 0.002; η_p_^2^ = 0.255) (see Figure 3b). Post-hoc analysis of the significant interaction showed a significant decrease in SCL values from t1 to t2 only in the accompanied group (t = 4.212, p < 0.001), whereas no such difference was obtained in the non-accompanied group (t = −0.569, p = 0.579). Moreover, groups did not differ in their t1 values (p = 0.675) neither in their t2 values (p = 0.085).

#### Mood

**Figure 4:**
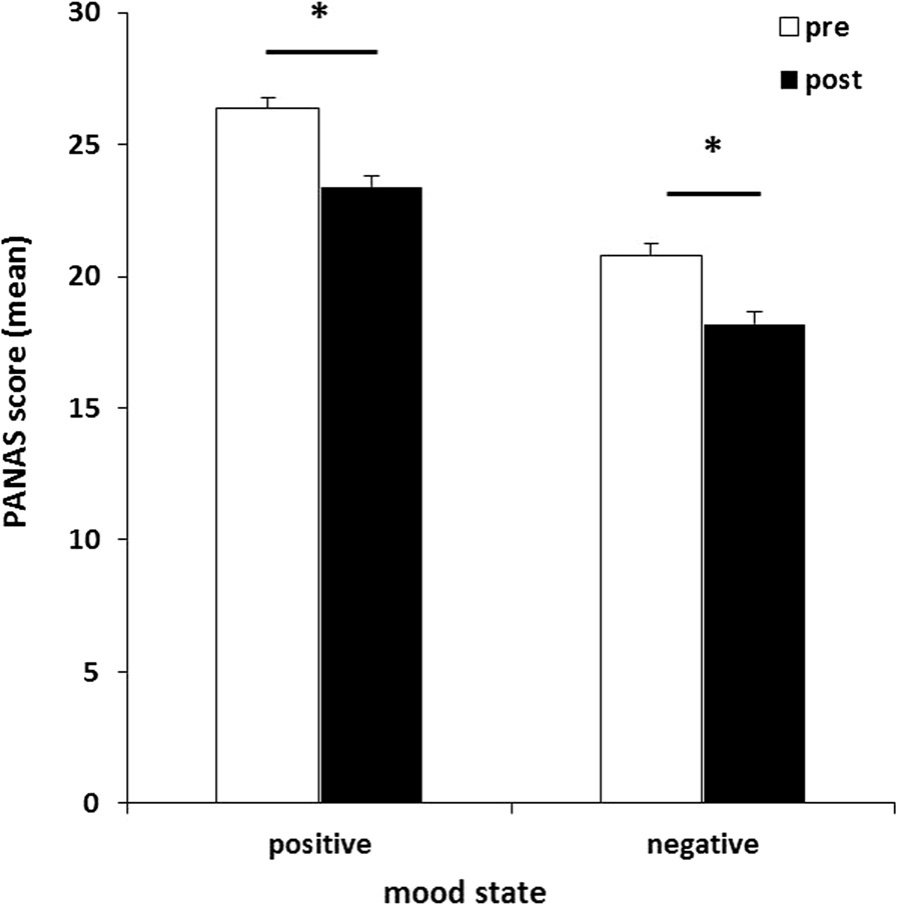
**Mean scores of positive and negative affect scale showing higher positive as well as negative scores immediately before compared to post fetal MRI examination for all subjects.** Significant differences are marked with an asterisk.

Changes in mood were analyzed using a rmANOVA with the within-subject factor time (pre and post MRI) and the between-subjects factor group (with or without accompanying person). For positive affect (PA, F(1,58) = 23.702, p < 0.001, (η_p_^2^ = 0.290)) as well as for negative affect (NA, F(1,58) = 10.616, p = 0.002, (η_p_^2^ = 0.155)) a main effect of time was revealed (see Figure 4). No significant group effect (PA: F(1,58) = 0.315, p = 0.577; NA: F(1,58) = 1.331, p = 0.253) and no time-by-group interaction (PA: F(1,58) = 0.941, p = 0.336; NA: F(1,58) = 0.821, p = 0.369) emerged.

Regarding the self rating of fear, a rmANOVA with time (t1, t2) and group as factors showed no significant time effect (F(1,58) = 2.815, p = 0.099), no significant group effect (F(1,58) = 0.311, p = 0.579) but a marginally significant time-by-group interaction (F(1,58) = 3.916, p = 0.053, η_p_^2^ = 0.063). Explorative post-hoc analysis showed that only in the accompanied group a significant reduction in fear ratings was apparent (p = 0.006), while no such decrease occurred in the non-accompanied group (p = 0.852).

Analysis of covariance including age as covariate (r = −0.292; p = 0.024) with group (with or without accompanying person) as fixed factor revealed no significant effect for depressive symptoms (BDI, F(2,57) = 2.795, p = 0.069) as well as for multivariate analysis of variance for state-anxiety (STAI, F(1,58) = 0.444, p = 0.508) or trait-anxiety (STAI, F(1,58) = 0.006, p = 0.939).

### Diagnoses

According to the diagnosis participants were divided into suspected but not confirmed fetal malformation on ultrasound (group 1, n = 10), fetal pathology compatible with survival (group 2, n = 35) and fetal pathology probably not compatible with survival (group 3, n = 15). Groups did not differ in gestational week (F(2,57) = 0.573, p = 0.567) but we observed a significant group difference in age (F(2,57) = 5.095, p = 0.009) with group 1 being significantly older than both other groups (1 vs. 2: p = 0.008; 1 vs. 3: p = 0.001), while group 2 and 3 did not differ (p = 0.703). See Table 4 for details.

#### Cortisol

**Figure 5:**
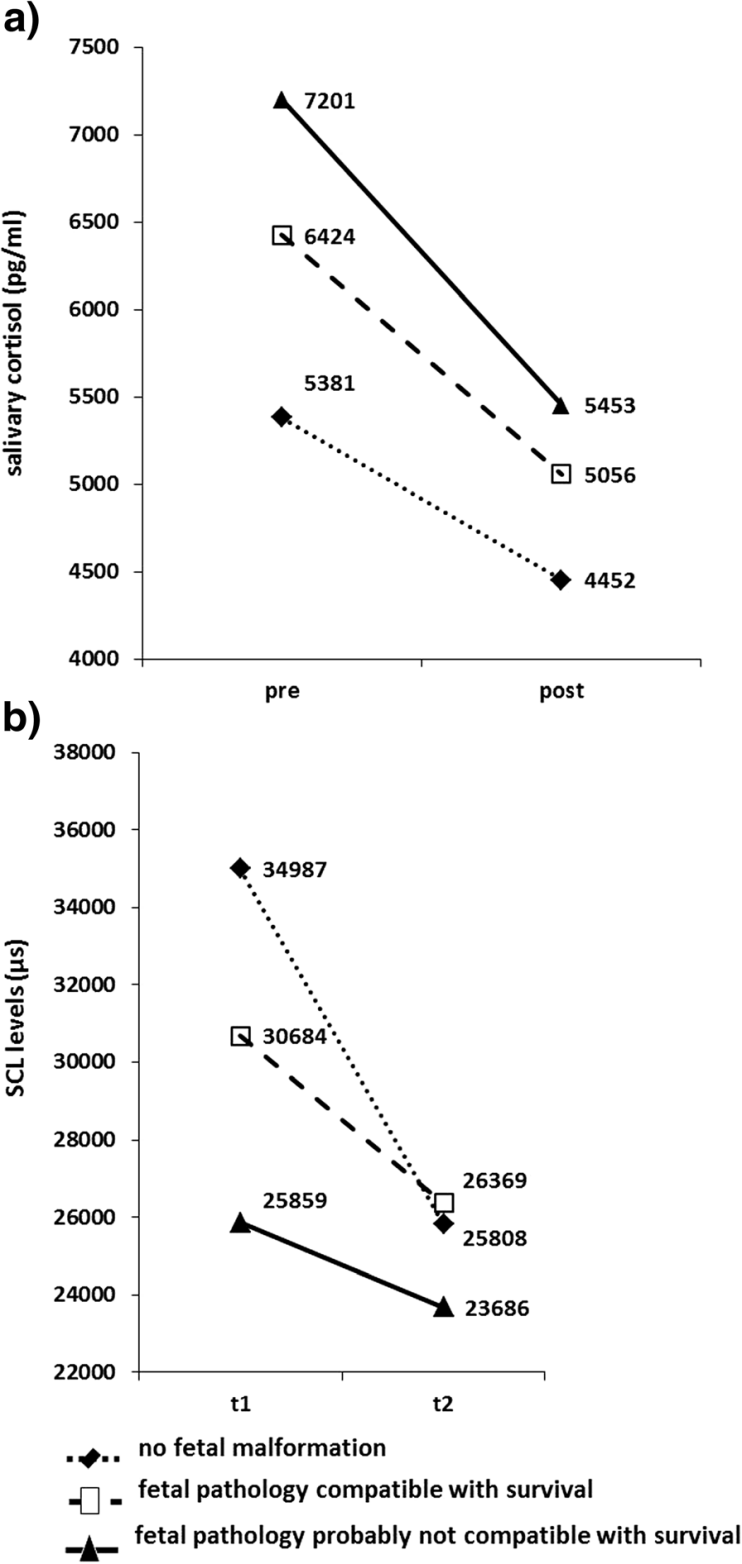
**Stress measures cortisol and skin conductance measures before and after fetal MRI depending on severity of diagnosis. a)** Cortisol levels immediately before (pre) and after (post) fetal MRI examination for women grouped by severity of diagnosis. All cortisol levels were reduced after MRI examination, with the best effect for the subgroup in which the fetal pathology was probably not compatible with survival. **b)** Skin conductance levels (SCL, in μs) of the first 5 minutes (t1) and last 5 minutes (t2) of fetal MRI examination of women grouped by severity of diagnosis. All SCL levels were reduced after MRI examination, with the best effect for the subgroup with no fetal malformation.

Analysis of covariance (including age) for repeated measurements with the within-subject factor time (pre and post MRI) and the between-subject factor diagnosis (groups 1, 2, 3) revealed no main effect of time (F(1,52) = 0.641, p = 0.427) as well as no significant diagnosis effect (F(2,52) = 0.835, p = 0.440). Moreover, no time-by-diagnosis interaction between severity of diagnosis on cortisol measures was obtained (F(2,52) = 1.571, p = 0.218) (see Figure 5a).

#### SCL

RmANOVA with time as within-subject factor (t1, t2) and diagnosis as between-subjects factor (groups 1, 2, 3) revealed a significant time effect (F(1, 32) = 8.463, p = 0.007, η_p_^2^ = 0.209) indicating lower levels at t2, no significant effect of diagnosis (F(2, 32) = 0.521, p = 0.599) or time-by-diagnosis interaction (F(2, 32) = 1.013, p = 0.375) (see Figure 5b).

#### Mood

Analysis of variance for repeated measurements with the within-subject factor time (pre and post MRI) and the between-subjects factor diagnosis revealed a significant effect of time for positive (PA, F(1,57) = 18.549, p < 0.001, η_p_^2^ = 0.246) as well as for negative (NA, F(1,57) = 9.207, p = 0.004, η_p_^2^ = 0.139) mood, with significantly lower positive and lower negative mood after MRI. However, no significant diagnosis effect (PA: F(2,57) = 0.126, p = 0.882; NA: F(2,57) = 0.357, p = 0.701) and no time-by-diagnosis interaction (PA: F(2,57) = 0.185, p = 0.832; NA: F(2,57) = 0.125; p = 0.883) emerged.

Regarding the self-rating of fear, no significant time effect (F(1,57) = 2.738, p = 0.104), no significant diagnosis effect (F(1,57) = 0.024, p = 0.976) and no significant time-by-diagnosis interaction (F(1,57) = 0.842, p = 0.436) emerged.

Multivariate analysis of variance with diagnosis as fixed factor revealed no main effect of severity of diagnosis for state anxiety (F(2,57) = 0.308, p = 0.736) or trait anxiety (F(2,57) = 0.754, p = 0.475). Moreover, no significant effect of diagnosis on depression scores (F(3,56) = 2.395, p = 0.078) was obtained.

### Stress regulation and coping strategies

Using the positive strategies (POS) of the SVF 120, females were distributed into three groups of stress coping strategies based on their percentile ranks: below average (PR = 0-24, n = 11), average (PR = 25-75, n = 30) and above average (PR = 76-100, n = 19) usage. Groups neither differed in age (F(2,57) = 0.129, p = 0.879) nor gestational week (F(2,57) = 0.083, p = 0.920). See also Table 4 for details. Due to fewer data sets of SCL-measurements and subsequent distribution of subjects, only two groups were built for SCL-levels: average (PR = 25-75) and above average (PR = 76-100).

#### Cortisol

Regarding cortisol levels, rmANCOVA with age as covariate and time as well as strategy group as factors revealed no significant time effect (F(1,48) = 0.003, p = 0.955), no significant strategy group effect (F(2,48) = 1.402, p = 0.256) and no significant time-by-group interaction (F(2,48) = 0.094, p = 0.910).

#### SCL

**Figure 6:**
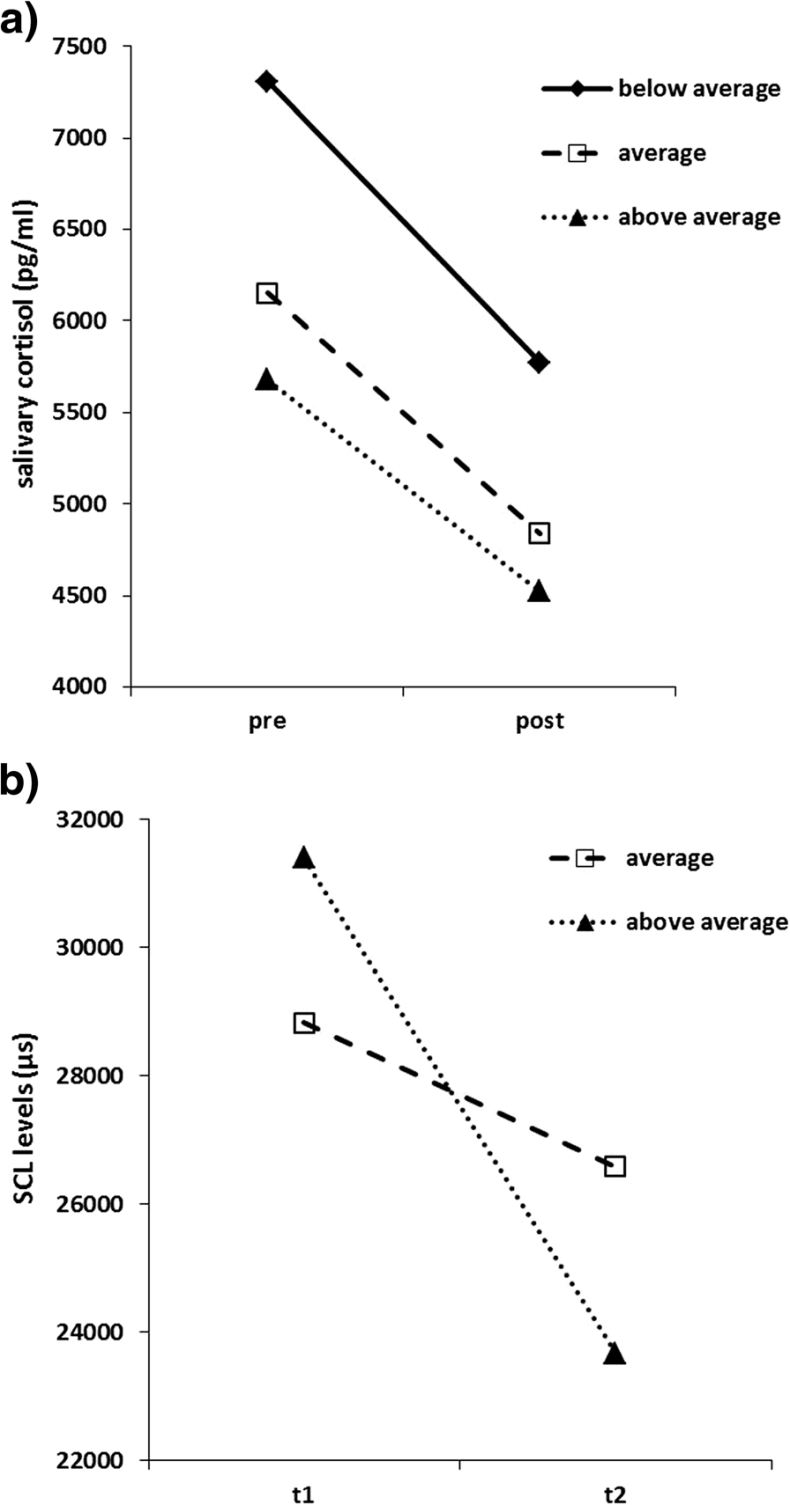
**Stress measures cortisol and skin conductance measures before and after fetal MRI depending on stress management strategies. a)** Cortisol levels immediately before (pre) and after (post) fetal MRI examination for women grouped by characteristics of their stress management strategies. All cortisol levels were reduced after MRI examination. **b)** Skin conductance levels (SCL, in μs) of the first 5 minutes (t1) and last 5 minutes (t2) of fetal MRI examination of women grouped by characteristics of their stress management strategies. Women with above average strategies were able to relax the most during the MRI examination.

RmANOVA with time as within-subject factor and strategy group as between-subjects factor revealed a significant time effect (F(1, 26) = 7.496, p = 0.011, η_p_^2^ = 0.224) but no significant group effect (F(1, 26) = 0.001, p = 0.972), and no significant time-by-group interaction (F(1, 26) = 2.265, p = 0.144) (see Figure 6b).

Regarding emotional states a multivariate analysis of variance with group as fixed factor showed no significant differences concerning positive (PA; F(2,57) = 1.310, p = 0.278) and negative (NA; F(2,57) = 0.281, p = 0.756) mood.

Regarding the self-ratings of fear, neither a significant time effect (F(1,57) = 2.688, p = 0.107), nor a significant group effect (F(2,57) = 1.485, p = 0.235) or time-by-group interaction (F(2,57) = 0.242, p = 0.786) emerged.

However, groups differed significantly in their BDI scores (F(3,52) = 4.031, p = 0.023, η_p_^2^ = 0.124). Bonferroni corrected post-hoc analysis showed a significant difference between women with above-average (group 3) and women with average stress coping strategies (group 2), obtaining lower depressive symptoms in women with above-average strategies (p = 0.038). All other comparisons remained not significant (1 vs. 2: p = 1.000; 1 vs. 3: p = 0.081).

To determine the impact of stress coping strategies on state- and trait-anxiety an analysis of variance with group (below average, average and above average) as fixed factor was performed. No significant influence of stress coping strategies on state- (F(2,53) = 1.196, p = 0.311) or trait-anxiety (F(2,53) = 2.083, p = 0.135) was obtained.

## Discussion

This study set out to offer a more thorough look on the emotional and physiological responses to fetal MRI in pregnant women by investigating stress levels examining three main factors, 1) accompanying person, 2) severity of diagnosis, and 3) stress management strategies used by the pregnant women.

### Stress response: Cortisol vs. Skin conductance levels (SCL)

We decided to assess salivary cortisol levels and skin conductance to measure different stress layers of response. The SAM-system reacts immediately after stress exposure and may therefore be referred to as the fast reacting pathway, as activation of this system directly leads to the release of catecholamines such as adrenalin and noradrenalin. In further consequence this release is followed by physical reactions like increase of heart rate, blood pressure, and changes in electrodermal activity [27]. Complementary to the SAM-system, the activation of the HPA-axis represents the slower stress response reaction and leads to the secretion of cortisol. The peak level of cortisol is reached approximately 21–40 minutes after the onset of a stressor [7]. Due to that complementary relationship we included both, the fast as well as the slow stress reaction response in our study.

### Accompanying person

Thirty-five of our female participants were accompanied by their partner, whereas 25 females were without partner or any other relevant person. Data analysis showed that for SCL values a significant group-by-time effect was observable indicating lower stress values in the last five minutes of MR scanning only in the accompanied group. In the non-accompanied group SCL levels showed no significant decrease over time but stayed relatively high throughout the experiment. We speculate that this significant decrease in women who know that someone personally relevant was waiting next to the MR scanner in the same room seems to have a comforting and positive effect on the stress response. Interestingly for cortisol we did not see such an interaction with group, only a time effect was apparent, with lower cortisol after the MR measurement. This was true for both groups.

Moreover, we also observed a significant effect of accompanying person on self-ratings of fear which were significantly lower at the end of the MR exam only in the accompanied group. Again, the single group did not show a decrease in fear level.

The investigation of how accompaniment influences psychological and physiological well-being is frequently performed in children undergoing medical examinations (e.g., [28,29]; for review). However, little is known on how accompaniment influences stress responses in adults undergoing a critical medical examination. In a previous study by Leithner et al. [6], the investigated women suggested to focus on the routine presence of the partner as an improvement in the procedure of fetal MRI. These results are in line with our data since we found decreased stress and fear levels in the accompanied group only.

### Severity of diagnosis

According to the severity of fetal diagnosis we split our sample into three groups: no fetal malformation but risk (group 1, n = 10), fetal pathology compatible with survival (group 2, n = 35) and fetal pathology probably not compatible with survival (group 3, n = 15). Interestingly and against our expectations, we did not see any significant impact of severity of diagnosis on stress responses. All three groups showed a decline in cortisol and a significant decrease of SCL levels over time, but no group-specific effect was detected. This was also true for mood and fear ratings.

Notably, Leithner and colleagues [4] observed a significant effect of severity of the referral diagnosis on anxiety levels before MRI. Anxiety measures showed a linear increase with pathology, indicating lowest anxiety in at-risk females and highest anxiety levels in females diagnosed with fetal malformation not compatible with survival. Interestingly, after the MRI exam the authors reported similar anxiety levels and thus a significant decline in all three groups. As the authors themselves state, information deficits about the procedure and the course of fetal MRI particularly in women with poor fetal prognoses negatively influence emotional and stress responses. In the present sample, 80% of the investigated females reported that they were intensively informed. Importantly, in the more severe groups (fetal pathology compatible or not compatible with survival) almost 90% of the females said they were informed, only in the at-risk group 40% were not informed. Thus, our findings support previous assumptions that information about the procedure and course of the medical examination helps pregnant women to cope with the stressful situation [30].

It would have been of great interest to compare maternal stress response after confirmation or exclusion of the fetal diagnosis after the MRI investigation. As patients are informed about the diagnosis of the MRI examination not immediately after the procedure but by their referring physician, this was not possible in the reported study, but should be investigated in future studies.

### Stress regulation and coping

Based on their profile of using positive stress coping strategies (POS) of the SVF 120, we distributed our sample to three groups: below average (PR = 0-24, n = 11), average (PR = 25-75, n = 30) and above average (PR = 76-100, n = 19) usage. Similar to the data on severity of diagnosis, we only observed an effect of time, with lower cortisol and significantly lower SCL levels after MRI across all groups.

Interestingly, groups differed in their self-rating of depressive symptoms, with the females with above-average scores reporting significantly less depressive symptoms than females with average coping strategies. Notably, groups did not differ in percentage of informed vs. not informed females or severity of diagnosis – both factors were distributed evenly across the three stress management groups. The experience of stress can be altered cognitively by relying on cognitive regulation strategies in order to decrease or down-regulate negative subjective experiences [31-33]. Moreover, Leithner et al. [6] reported that in their study, the investigated females tried to down-regulate their distress and fear by distraction or by thinking “agreeable thoughts”. Being able to down-regulate ones sensations and feelings might not only be a positive option for patients undergoing critical medical exams but also before in order to decrease depressive symptoms that might arise due to the medical situation or the anticipation of the medical exam.

## Conclusions

Taken together, SCL data and fear self-rating indicate a significant positive effect of accompanying person on stress and emotional reactions in pregnant women undergoing fetal MRI. Moreover, our data support previous findings of a positive effect of information on the procedure and course of fetal MRI on stress and emotional responses in high-risk pregnant females. And, introducing or supporting emotion regulation strategies might help to decrease depressive symptomatology in these patients and thus might help to improve patient’s comfort.
